# Editorial: How school health and nutrition interventions are reshaping the global public health narrative

**DOI:** 10.3389/fpubh.2026.1804746

**Published:** 2026-03-18

**Authors:** Lesley Drake, Samrat Singh, Boitshepo Giyose, Donald A. P. Bundy

**Affiliations:** 1 School of Public Health, Imperial College London, London, United Kingdom; 2 Food and Agriculture Organization, Johannesburg, South Africa; 3 London School of Hygiene and Tropical Medicine, London, United Kingdom

**Keywords:** children, health, nutrition, policy, school

This new collection of articles on school health and nutrition (SHN) programs recounts the extraordinary story of how these programs have emerged as a nearly ubiquitous, country-led investment in the health, education, and wealth of future generations.

We set out to explore contemporary thinking and research on school health and nutrition programs, resulting in this Research Topic of 14 studies that highlight the diversity of SHN interventions and their role in influencing public health thinking across a wide range of topics, including WASH, malaria prevention, brain health, and nutritional education.

Since the 2008 financial crisis, and with a more recent push from the school closures during the COVID pandemic, there has been a growing realization of the central role of SHN in creating the human capital on which the wealth of nations depends. This recognition has resulted in a paradigm shift in government thinking, with SHN programs becoming the world's largest social safety net and a nearly ubiquitous, government-funded investment in future generations. Today, almost all countries view investing in the health and well-being of schoolchildren and adolescents as necessary public policy and an essential counterpart to free, compulsory, universal education.

In this editorial, we discuss how the 14 contributions to our new Research Topic explore the evolutionary trajectory of SHN programs by describing the emergence of four milestone principles: the centrality of a multisectoral approach; the key role of human capital creation; the salience of school meals as an organizing principle; and the recognition that healthy behaviors established at school age have lifelong consequences for both human and planetary health.

## The first milestone: recognizing the need for multisectoral engagement

At the start of the 20th century, many countries implemented some form of SHN program with a focus on a clinical approach to the health of schoolchildren. The majority of programs relied on mobile health sector teams traveling to schools, with challenging logistical and budgetary constraints regarding scale and sustainability.

**Figure 1:**
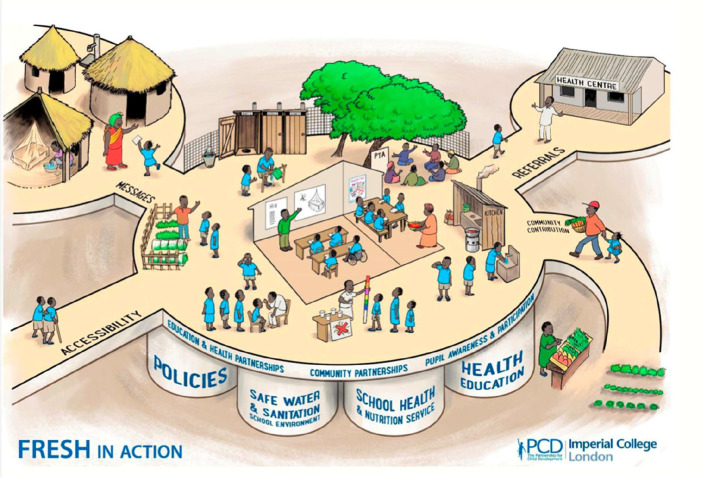
Fresh framework- componets and pathways.

The World Education Forum Conference in Dakar in 2000 introduced a new line of thought: if healthy children learn better and sick children may not even come to school at all, could SHN programs influence education sector objectives in addition to the health sector ones? Also, given that there are far more teachers than nurses and more schools than clinics, could programs be redesigned so that the health and education sectors work together? The launch of the FRESH (Focusing Resources on Effective School Health) framework at Dakar was a turning point (1). This new way of thinking saw key agencies, notably UNESCO, UNICEF, WHO, the World Bank, and other partners, including PCD and NEPAD/AUDA, join forces to promote the FRESH concept, which sees schools as platforms for four key interventions: school-based health policies; access to safe water and sanitation; health and nutrition services; and skills-based health education. These interventions are supported by strong partnerships at all levels and with clearly defined roles and responsibilities for both the health and education sectors (see Figure 1 for a contemporary cartoon illustrating the interconnectedness of all these interventions around one school setting).

All the articles in our Research Topic, to a greater or lesser extent, underline the importance and impact of this engagement.

## The second milestone: recognizing the role of SHN programs in human capital investment

The importance of investing in health during the first 1,000 days of life has long been recognized and needs no further emphasis here. However, an unfortunate consequence of this hyper-focus is that little to no structured support is offered to children after day 1,001, and the health of schoolchildren and adolescents is largely unsupported during a period of extraordinary developmental changes, including puberty. During this time, they are also expected to learn and become educated. Even in the most basic economic terms, this made no sense. The case was eloquently made in the World Bank's *Disease Control Priorities Edition 3*, especially volume 8, the *Health and Development of School Children and Adolescents. The book argued* that the safety net needed to be widened to include these age groups, and stronger, more systematic, and more evidence-driven interventions and support structures were needed during the “next 7,000 days” (2). This concept of supporting a child for 8,000 days was new: during the especially vulnerable first 1,000 days of life and then throughout the 7,000 days of middle childhood and adolescence when children are educated, develop into adults, and acquire habits and skills that shape the rest of their lives.

Importantly, analyses have shown a major historical disparity in resource allocation, research attention, and consequently understanding of the health and nutrition issues, with a historically much stronger focus on the first 1,000 days. This is now moving toward a more balanced and appropriate response, and two studies in this Research Topic, by Irizarry et al. and Schultz et al., reflect on the implications of these disparities.

Today, there is widespread recognition of the importance of health and wellbeing for learning and educational outcomes. This idea has been most recently and clearly articulated by UNESCO in key publications (3, 4). A recent World Bank report on human capital has called for coordinated action across neighborhoods, workplaces, and homes, and across health, social protection, agriculture, transport, water, and sanitation (5). Taken together, these concepts provide the strongest argument for SHN programs as a key investment in the next generation of societies.

## The third milestone: recognizing the importance of school meals as part of SHN programs

The social shocks resulting from the Food, Fuel, and Finance crisis of 2008 brought new attention to school meals, which emerged as a key response by governments worldwide. This new role for school meals was described in a 2009 World Bank publication entitled “Rethinking School Feeding,” which heralded a decade of growth in these programs, notably including new national programs in China and Russia (6).

This movement gained further momentum when schools closed during the COVID pandemic, leading countries worldwide to recognize the importance of schools, school meals, and SHN programs in supporting future generations. In 2021, the School Meals Coalition (SMC) was launched. This has become one of the most notable multilateral initiatives that have arisen from the pandemic. The SMC is a country-led initiative with 112 member states, and 150 partner organizations, currently chaired by the Presidents of Brazil, Finland, and France. The primary mandate of the coalition is to help its member countries drive actions to urgently improve and scale up school meal programs and complementary SHN actions globally.

Many national programs were already well-established; for example, there are universal school meal programs in Brazil, Finland, India, Japan, South Korea, and Sweden. However, there has also been a dramatic increase in coverage of more than 80 million children from other programs in the 4 years since the SMC was established, with the fastest growth where the need was greatest. For example, some 87 million children were fed in Africa in 2024, up from 38 million in 2013. Furthermore, several countries are launching national programs for the first time, including Canada, Denmark, the Netherlands, Indonesia, and Ukraine.

Today, school meal programs still represent the most extensive social safety net programs in the world, covering 169 countries and feeding over 466 million children every school day. Global investment in school meal programs now stands at US$84 billion annually, with 99% coming from domestic budgets. School meals are now the largest component of SHN programs worldwide.

In response to this demand, the SMC created the Research Consortium (RC) initiative to gather evidence supporting policies that emphasize the cost-efficiency of school meals and SHN programs. The RC brings together global academia, think tanks, and research networks to inform evidence-based decision-making around SHN programs. A summary of the work of the RC is documented by Schultz et al. in this Research Topic.

Analysis has shown that funding and supporting school meals programs provide the financial platform to enable the implementation of less costly, synergistic SHN interventions that might not be as cost-efficient if delivered individually.

## The fourth milestone: recognizing that well-designed SHN programs can make a crucial contribution to promoting planetary health

SHN programs, in particular, the evolution of Home-Grown School Feeding, are resulting in one of the most significant policy shifts in global public health by incorporating multiple dimensions of planetary health into policy and practice, including the climate change narrative (7). At COP28, the Research Consortium of the SMC released a white paper on *Planet-Friendly School Meals*, highlighting the potential of school feeding programs to promote sustainable food systems, improve nutrition, and combat climate change (8). Climate-smart menus and local sourcing can help reduce food system-related GHG emissions, positioning school meals as an important lever for greener and more sustainable food systems. In addition, well-designed school meal programs can help children establish lifelong dietary preferences for environmentally sustainable foods.

COP28 saw many countries committing to expanding these programs, with initiatives focused on integrating them into climate finance and adopting sustainable procurement practices. At the recent UNFSS +4 in Ethiopia, a specific convening was held on the role of school meals in agroecology and regenerative agriculture, highlighting operational pathways between ecological services and public health outcomes. For the first time, the Ministry of Agriculture and the Ministry of Environment in countries such as Rwanda, Ghana, Nigeria and Kenya have been included in the operational discussion around SHN programs and school meals.

By providing children with models of sustainable and healthy foods at school, and supporting this with food education, the world is changing children's relationship with food and helping develop lifelong dietary preferences, as emphasized in the latest report of the EAT-Lancet Commission (9). These interventions simultaneously promote personal health, planetary health, and agroecological/regenerative approaches to food production.

In conclusion, we set out to develop this Research Topic to explore contemporary thinking and research around SHN. We invited contributors from a wide range of disciplines, sectors, and organizations, resulting in a Research Topic that highlights the diversity of SHN interventions and their role in influencing public health thinking across a wide range of topics, all of which support the four key milestones in the evolution of SHN.

This Research Topic highlights that these four key paradigm shifts have underpinned the evolution of SHNPs, transforming them from small-scale, health sector-driven interventions to critical investments in human capital and a key part of the global public health development agenda.

By their very nature, SHN programs are helping to develop a new generation of climate-aware and educated minds, a truly global phenomenon.

## References

[1] Focusing Resources on Effective School Health: A FRESH Start to Enhancing the Quality and Equity of Education. Focusing Resources on Effective School Health (FRESH) Series. Washington DC: World Bank Group (2000).

[2] Bundy D de Silva N Horton S Jamison DT Patton GC editors. Child and Adolescent Health and Development. Disease Control Priorities. 3rd ed. Vol. 8. Washington, DC: World Bank (2017). doi: 10.1596/978-1-4648-0423-6_ch1

[3] UNESCO UNICEF WFP. Ready to learn and thrive: School Health and Nutrition Around the World. Paris: UNESCO, UNICEF, WFP (2023).

[4] UNESCO GPE Research Consortium for School Health and Nutrition UNESCO UNESCO Chair on Global Health and Education UNICEF WFP The The World Bank and WHO. Integrating Health and Well-being Into Education Policy and Planning: A Handbook. Paris: UNESCO (2025).

[5] Alaka H Schady N Silva J editors. Building Human Capital Where It Matters: Homes, Neighborhoods, and Workplaces. Washington DC: World Bank (2026).

[6] Bundy D Burbano C Grosh M Gelli A Jukes M Drake L. Rethinking School Feeding: Social Safety Nets, Child Development and the Education Sector. Washington DC: The International Bank for Reconstruction and Development (2019).

[7] Singh S Jordan I Hunter D Milani P Muthoni P Borelli T. Promoting climate-resilient agriculture and food security through school feeding. Lancet Planetary Health (2026). doi: 10.1016/j.lanplh.2025.101414 41720118

[8] Pastorino S Backlund U Bellanca R Hunter D Kaljonen M Singh S . Planet-friendly school meals: opportunities to improve children's health and leverage change in food systems. Lancet Planet Health. (2025) 9:11. doi: 10.1016/S2542-5196(24)00302-4 39571585

[9] Rockström J Thilsted SH Willett WC Gordon LJ Herrero M Hicks CC . The EAT–*Lancet* Commission on healthy, sustainable, and just food systems. Lancet. (2025) 406:1625–700. doi: 10.1016/S0140-6736(25)01201-2 41046857

